# Supplementary material for: Content-rich biological network constructed by mining PubMed abstracts
Source: BMC Bioinformatics. 2004 Oct 8;5:147. doi: 10.1186/1471-2105-5-147 (PMC528731; doi:10.1186/1471-2105-5-147)
Supplement: Additional File 2 — The original results of the above study (non-essential files are deleted to keep the file size under the limit set by BMC bioinformatics). [file 1471-2105-5-147-S2.bz2 › chilibotAdditionalFile2/dip05/270ID2169352E1090/html/NR3C1_JUN.html]

 


 **NR3C1** and **JUN** 
  
Found 213 abstracts in PubMed, retrieved 05.  
 

 What does Google say? 
 PDF only 
| .edu only 

---

**Interactive relationship** (e.g. stimulation, inhibition, etc)

**Inhibitory relationship**- Regardless of the AP 1  [ **JUN** ]  complex s composition, glucocorticoid receptor  [ **NR3C1** ]  activation inhibits AP 1  [ **JUN** ]  and prevents apoptosis.  Ref: 12766089 Invest Ophthalmol Vis Sci, 2003
**Neutral relationship**

**Non-interactive relationship** (e.g. studied together, co-existance, homology, etc.)

- These results suggest that AP 1  [ **JUN** ]  proteins and glucocorticoid receptor  [ **NR3C1** ]  regulate transcription of the GnRH R promoter in a heterologous system.  Ref: 12644302 Mol Cell Endocrinol, 2003
